# Lightweight Detection Method for X-ray Security Inspection with Occlusion

**DOI:** 10.3390/s24031002

**Published:** 2024-02-04

**Authors:** Zanshi Wang, Xiaohua Wang, Yueting Shi, Hang Qi, Minli Jia, Weijiang Wang

**Affiliations:** 1 School of Integrated Circuits and Electronics, Beijing Institute of Technology, Beijing 100081, China; wangzanshii@163.com (Z.W.); xh_wong@bit.edu.cn (X.W.); qihangbit@163.com (H.Q.); 7420220059@bit.edu.cn (M.J.); 2 Science and Technology on Millimeter-Wave Laboratory, Beijing Institute of Remote-Sensing Equipment, Beijing 100854, China; ytshical@163.com

**Keywords:** object detection, YOLOv8, X-ray security inspection, lightweight model, deep learning

## Abstract

Identifying the classes and locations of prohibited items is the target of security inspection. However, X-ray security inspection images with insufficient feature extraction, imbalance between easy and hard samples, and occlusion lead to poor detection accuracy. To address the above problems, an object-detection method based on YOLOv8 is proposed. Firstly, an ASFF (adaptive spatial feature fusion) and a weighted feature concatenation algorithm are introduced to fully extract the scale features from input images. In this way, the model can learn further details in training. Secondly, CoordAtt (coordinate attention module), which belongs to the hybrid attention mechanism, is embedded to enhance the learning of features of interest. Then, the slide loss function is introduced to balance the simple samples and the difficult samples. Finally, Soft-NMS (non-maximum suppression) is introduced to resist the conditions containing occlusion. The experimental result shows that mAP (mean average precision) achieves 90.2%, 90.5%, 79.1%, and 91.4% on the Easy, Hard, and Hidden sets of the PIDray and SIXray public test set, respectively. Contrasted with original model, the mAP of our proposed YOLOv8n model increased by 2.7%, 3.1%, 9.3%, and 2.4%, respectively. Furthermore, the parameter count of the modified YOLOv8n model is roughly only 3 million.

## 1. Introduction

X-ray security inspection is the main method of security inspection at passenger stations [1]. An X-ray security check is the process of placing luggage into a penetrating X-ray security machine, which generates X-ray images on the terminal device in real-time. Security personnel need to identify whether there are prohibited items in the luggage within a short period of time during conveyor belt transition. The types and positions of objects in the luggage are complex and varied, and items inside packages can also overlap with each other, causing obstruction and making it difficult to identify them in a short period of time. Manual inspection requires the highly focused attention of the detection personnel; relying solely on manual security inspection will inevitably be affected by human physical capabilities and fatigue. The use of computer vision and mapping technologies to assist staff at X-ray security inspection stations can effectively reduce the problem of false or missed inspections caused by a lack of experience or the physical fatigue of security inspectors [2]. The huge workload and intensity of X-ray security inspection make security inspection assisted by computer vision technology an inevitable trend. 

Traditional machine-learning-based X-ray image detection methods adopt manual features and classifiers for classification, while positioning information is obtained through sliding window methods [3]. The bag of visual words method is commonly used in traditional X-ray image detection. Yang et al. [4] studied the method of detecting and classifying liquids by combining EDXRD (energy dispersive X-ray diffraction) with linear discriminant analysis and principal component analysis. Wang et al. [5] proposed a classification method based on the Tamura texture feature and random forest to extract features and classify four types of contraband. The classifier uses random forest, AdaBoost, decision tree, and the Bayesian network for comparison, and the results show that the Tamura feature combination random forest classifier has the best classification performance. 

In recent years, deep learning methods based on convolutional neural networks have achieved breakthrough development, and the task of applying them to X-ray security for target detection has also been widely developed. There are two types of target detectors, a one-stage detector [6,7] and a two-stage [8,9] detector. The difference between the two is that there is no region proposal generation in a one-stage detector algorithm. The accuracy of the one-stage detector is lower than that of the two-stage detector, but the detection speed of the one-stage detector is faster. Akcay et al. [10] first introduced deep learning into the baggage classification detection of X-ray images and used transfer learning to apply the AlexNet network to the research of X-ray image baggage classification. Fang et al. [11] proposed a few-shot SVM-constraint threat detection model, and achieved 85.7%, 82.8%, and 84.5% bAP (base average precision), and 88.4%, 88.4%, and 85.1% on 10-shot and 30-shot samples under three class divisions, respectively. Zhang et al. [12] proposed the XMC R-CNN model, which uses the X-ray material classifier algorithm and the organic stripping and inorganic stripping algorithms to solve the problem of contraband detection in overlapping X-ray baggage images. Zhang et al. [13] improved the SSD (single-shot multibox detector) network by adding small convolutional asymmetric modules and multi-scale feature map fusion modules to enhance the detection performance for small targets. Sun et al. [14] proposed an algorithm based on SSD, which integrates MSA (multi-scale attention architecture) and MSE (multi-scale feature extraction) structures to eliminate redundant features and enrich the contextual information; the algorithm achieved a 7.4% mAP improvement on X-ray security images compared to original approach. Zhu et al. [15] proposed an attention-based multi-scale object detection network AMOD-Net for X-ray baggage security inspection; they designed a channel selection attention module to solve the problems of stacking and occlusion that were extant in the X-ray baggage image; they achieved 86.7%, 88.3%, and 66.9% mAP values for the Easy, Hard, and Hidden test sets of the PIDray [16] datasets, respectively. Compared to traditional machine learning, deep learning exhibits better detection performance.

In recent years, the YOLO series algorithm, which is a one-stage detector, has been updated and upgraded several times, and has also achieved some research results in X-ray security detection tasks. Guo et al. [17] changed its backbone network to a new backbone network composed of two darknets based on YOLOv3 and introduced a six-layer convolution feature enhancement module to enhance the detection effect for small targets in X-ray images. In the task of detecting concealed objects during security checks, Pang et al. [18] proposed an algorithm based on YOLOv3, which reaches a 95% mAP in PMMW (passive millimeter wave) contraband images. Yu et al. [19] improved the YOLOv4’s PANet (path aggregation network) module with deformable convolution and introduced the Focal-EIoU loss function. Furthermore, the improved model reached 85.51% mAP on SIXray [20] datasets. Song et al. [21] designed a small-sized YOLOv5 model incorporating the Stem and CGhost modules, and used the mix-up data augmentation method to learn the de-occluded ability. The mean accuracies of the improved algorithm reached 88.2%, 89.2%, and 70.5%, respectively, and improved by 2.5%, 1.2%, and 5.0%, respectively, on the three test sets of the PIDray datasets, respectively. Xiang et al. [22] designed a new network integrated MSA (multi-scale smoothed atrous) convolution and MCA (material-aware coordinate attention) module based on YOLOX algorithm, and introduced the SD-SIoU (shape decoupled SIoU) loss function for learning the prohibited items shape features. The improved model reached the accuracies of 92.92% and 91.1% on the OPIXray [23] datasets and SIXray datasets, respectively. Cheng et al. [24] designed a rotated target detection algorithm based on improved YOLOv7 to ignore the random placement of objects in baggage, achieving mean accuracies of 84.5%, 65.4%, and 49.2% on the three test sets of the PIDray datasets, respectively. Jing et al. [25] proposed an algorithm based on YOLOv7, which integrated an EFE (edge feature extractor) module and an MFE (material feature extractor) module.

The main research results in X-ray security detection based on YOLO algorithms were introduced above. Massively modified strategies consisted of applying attention mechanism modules or increasing receptive fields. Moreover, the modified methods can also focus on the enhancement of extracted image information to train the model to learn more feature information surrounding the X-ray contraband. The size of the same class of hazardous items can sometimes be vary greatly; for example, knives can be large or small. In the FPN (feature pyramid network) structure, the objects of different sizes will appear on feature maps of different scales. When a feature map matches an object, the information of feature maps from other layers will be ignored. The inconsistency of features at different scales is a major drawback of the feature pyramid, especially for one-stage detectors. YOLO series algorithms have been using the improved FPN structures since the third generation. The features of different layers are different, and roughly direct fusion may inevitably lose valuable information. Traditional feature fusion often involves simply overlaying or adding feature maps, such as using concatenate or shortcut connections. The contributions of feature maps obtained through different convolutions to the fusion feature maps are also different, so simply adding or overlaying them is not the best approach. X-ray security check images also have their own characteristics. The overlap and occlusion between items have always been an important issue in X-ray security inspection. Wei et al. [23] proposed a plug-and-play DOAM (de-occlusion attention module), which is motivated by different shape appearances, colors, and textures of variant-occluded objects. Limited by the information of collected images from the real world, there is an imbalance between the easy samples and the hard samples in the available X-ray datasets. Instances in which it is easy to extract and distinguish features in training may be particularly abundant; meanwhile, others may be very scarce, so it is difficult to support the learning needs of the model. Previous research on multi-scale feature extraction and fusion has not paid much attention to the contributions of features, and many improved models based on the YOLO series often fail to consider the shortcomings of the FPN structure in feature fusion. The existing models have poor detection performance for occluded scenes. In addition, a small amount of studies have resulted in models with too large a parameter count; this is not suitable for deployment in real devices. To address the above issues, we provide a new method for the X-ray detection of prohibited items.

The main contributions of this paper are as follows:

(1) In order to solve the problem of insufficient feature learning provided by the X-ray image datasets and the lack consideration for the contributions of features from different sources, ASFF architecture modules and a weighted concatenate algorithm were introduced to obtain finer and more comprehensive feature representation.
(2) To increase the attention paid to important information and suppress background noise, the CoordAtt module was embedded into the detection frameworks. The slide loss function was applied to solve the imbalance between simple samples and difficult samples. And we applied the Soft-NMS method to enhance the de-occluded ability of model.
(3) Based on the above algorithms, we proposed the lightweight, high-precision, and de-occluded YOLOv8n and YOLOv8m detection models. The improved YOLOv8n model already has superior detection performance, while the YOLOv8m model has higher accuracy. The comparative experiments results show that our models can achieve state-of-the-art detection accuracies on the PIDray and SIXray datasets.

## 2. Background

### 2.1. X-ray Security Inspection

In computer vision research on X-ray security inspection, there are three commonly used datasets: SIXray, OPIXray, and PIDray. There are many research results already published which have been derived on the basis of these three datasets. The SIXray dataset is a large dataset for prohibited item discovery in X-ray security inspection. The SIXray dataset consists of 1,059,231 X-ray images, mainly comprising five classes of object: knife, gun, wrench, pliers, and scissors. There are 8929 prohibited items in the dataset which are manually annotated. This dataset was created to enable study of the impacts of an imbalance between positive and negative samples and to create a testing environment which mimics a real-world scenario; in the real-world scenario, inspectors often aim to recognize prohibited items that appear in a very low frequency. Therefore, the dataset has a particularly large number of negative samples. The OPIXray dataset was the first dataset that has been specially designed for the detection of occluded prohibited items in X-ray security inspection. It focuses on a frequently encountered prohibited item, knives, and contains five knife classes comprising 7109 training images and 1776 testing images. In order to explore the impact of object occlusion degrees, the test set is further divided into three occlusion levels to enable a better understanding of the performance of the detectors.

**Figure 1:**
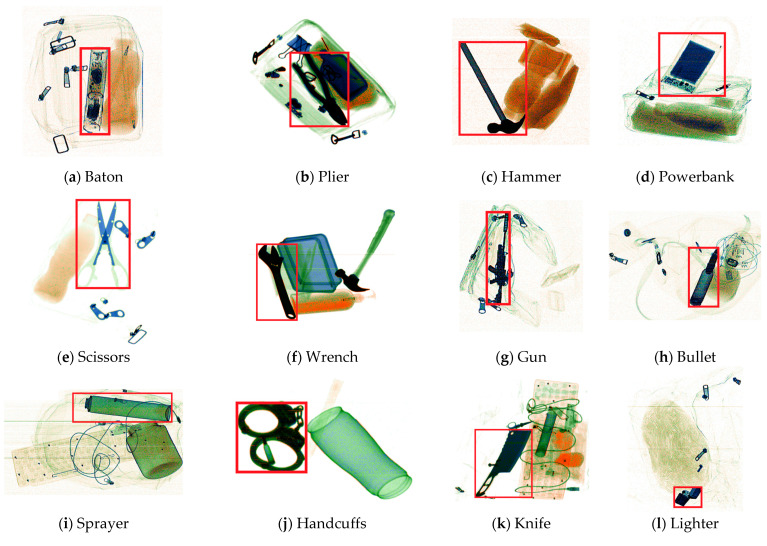

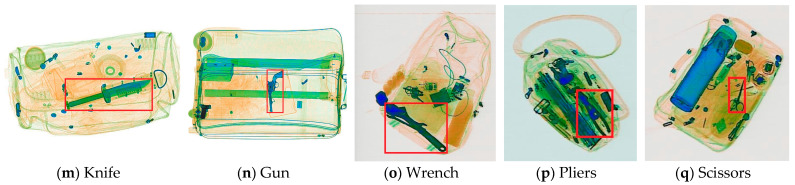
Examples of prohibited items from the PIDray dataset (**a**–**l**) and the SIXray dataset (**m**–**q**).

Due to the limitations of producing X-ray check data in the real world, the existing datasets have a limited data volume and limitations in the types of hazardous objects included. In the interest of moving toward a real-world practical application, Wang et al. [16] collected a large-scale prohibited-item-detection dataset named PIDray, which covers 12 common categories of prohibited items: gun, knife, wrench, pliers, scissors, hammer, handcuffs, baton, sprayer, powerbank, lighter, and bullet. Twelve types of hazardous articles from the PIDray dataset and five from the SIXray dataset are presented in Figure 1.

The PIDray dataset was collected in different scenarios such as airports, subway stations, and railway stations. The dataset was created to include three types of images in different sizes and colors, identified by three security inspection machines. The PIDray dataset contains 47,677 images; of these, approximately 60% comprises the training dataset, consisting of 29,457 images, and approximately 40% comprises the testing dataset, consisting of 18,220 images. In addition, in view of the difficulties encountered in detecting prohibited items, the test set was divided into three subsets: Easy, Hard, and Hidden. Specifically, the Easy level, with 9482 images, includes the images in the test set that only contain one prohibited item. The Hard level, consisting of 3773 images, includes images that contain multiple prohibited items. The Hidden level, which contains 5005 images, comprises images that contain intentionally hidden prohibited objects. Due to the large number of training images and various types of objects in dataset, the PIDray dataset and SIXray dataset were selected for evaluating the performance of the proposed method.

### 2.2. YOLOv8

In 2023, the YOLO series has been iterated to YOLOv8, and both YOLOv5 and YOLOv8 are from the same authors. The YOLOv8 algorithm is inherited and improved from previous versions. In the backbone, YOLOv8 still adopts the CSP concept, but the C3 modules in the YOLOv5 have been replaced by C2f modules to enhance the lightweight character. The C2f module has two convolutions and a spilt operation outside of CSP bottlenecks. The C2f module is designed based on the ideas of the C3 module and ELAN, allowing YOLOv8 to obtain richer gradient flow information while ensuring its lightweight character.

Moreover, YOLOv8 still uses the SPPF (spatial pyramid pooling-fast) module which was used in the YOLOv5 architecture. In the neck section, YOLOv8 still makes use of the PAN-FPN structure, so there will still be an inconsistency issue with feature information at different scales in YOLOv8. In the design of the detect section, YOLOv8 uses the decoupled head, which outputs classification and regression subsections through two headers, respectively. In terms of loss function, the YOLOv8 algorithm employs the BCE (binary cross entropy) loss function to represent classification loss. The regression branch of the loss function applies DFL (distribution focal loss) loss and CIoU (complete-IoU) loss. The total loss function is obtained by weighting the three losses with weight proportions. The total loss is defined as:

(1) $$L_{total}=h_{BCE}\cdot l_{BCE}+h_{CIoU}\cdot l_{CIoU}+h_{DFL}\cdot l_{DFL}$$

The $L_{total}$ is the total loss function value. The $h_{BCE}$ is the weight hyperparameter of the BCE loss function, $l_{BCE}$ is the BCE loss function value, and the other parameters represent these as the same principle. In terms of label assignment, YOLOv8 abandons the previous IoU matching or unilateral proportional assignment methods and instead uses the task-aligned assigner-matching method. Moreover, the YOLOv8 detection head is anchor-free, which is more conducive to learning multi-scale features than anchor-based detection heads [26].

## 3. Method

**Figure 2:**
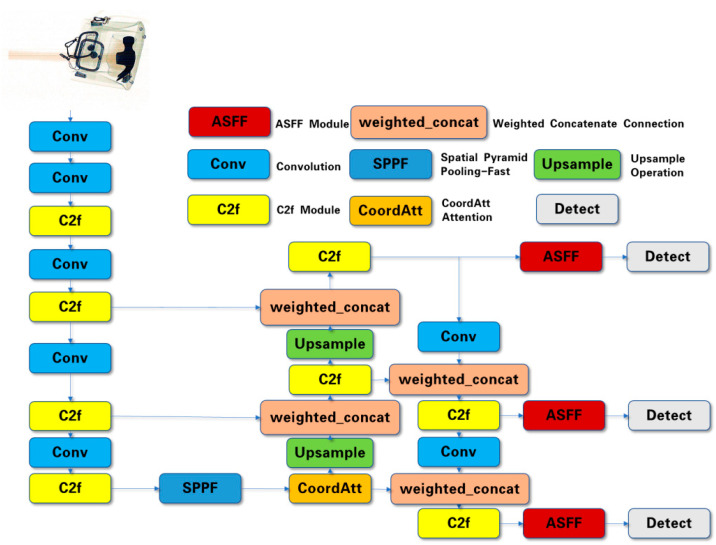
Improved network structure of YOLOv8.

The proposed method is a high-precision and de-occluded X-ray security detection method that has been built to deal with many problems in X-ray scanning. The method includes the following: feature fusion, an attention mechanism, a weighted modification of the loss function, and a post-processing algorithm for prediction. Firstly, after the SPPF module, an attention module is introduced in the network, which ensures that, after multiple convolution operations, the feature matrix focuses more on important information. Secondly, the ASFF module is embedded at the front of the detect head, and weighted concatenate connections are introduced to excavate more useful information from the X-ray images and improve the efficiency of multi-scale information utilization. Then, the slide loss function operation is applied in the BCE loss function, which implements extra focus on learning for difficult samples. Finally, the detection performance of occluded objects has been improved by the Soft-NMS algorithm. The improved network structure is shown in Figure 2.

### 3.1. ASFF

When using feature pyramids to detect objects, large instances are mapped to high-level feature maps, while small instances are displayed in low-level feature maps. When an instance of a certain feature layer belongs to a positive sample, it means that the corresponding areas on other feature layers will be considered to be backgrounds. The abovementioned contradiction between the different level features disturbs the gradient calculation during training, reducing the effectiveness of the feature pyramid. This is a common flaw in feature pyramids in single-stage target detectors. The ASFF [27] algorithm can solve this problem and increase the cognition of the features. The ASFF algorithm is an adaptive fusion strategy that achieves joint feature fusion, and it performs well in one-stage target detection. 

**Figure 3:**
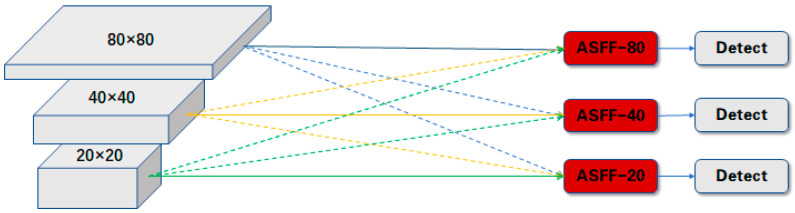
ASFF structure diagrammatic sketch.

There are three sizes of feature map outputs from the feature pyramid, which have the following sizes: 80 × 80, 40 × 40, and 20 × 20. After embedding the ASFF structure into the network, the process of inputting these feature maps into the detection layers is not like the one as shown in Figure 2, which is directly inputting them into the detection head through a single channel; the real input process is akin to that presented in Figure 3. The feature maps of each size will ultimately be input into three detection heads separately. Meanwhile, the features from the three scale sources are sent to each detection head, which are up-sampled, down-sampled, or unchanged to ensure a consistent, unified size; then, they are weighted separately. The dashed lines with arrows pointing downwards represent the subsampling process. On the contrary, the dashed lines with arrows pointing upwards represent the up-sampling process. Taking the input features to the detection head with size of 80 × 80 as an example: those feature maps which move into the detection head with a size of 80 × 80 are comprised of the sampled 40 × 40 and 20 × 20 feature maps from the feature pyramid, as well as the raw 80 × 80 feature map outputs from the feature pyramid; these are multiplied by their respective weights and summed, element by element, to obtain the input of ASFF-80 in the diagram. In Figure 3, the solid lines mean the unchanged delivery, and the dotted lines mean the sampling process. Different colors represent sources of different scales. The calculation process of ASFF-80 is represented by the following equation:

(2) $$ASFF-80=\alpha^{80}\cdot f^{80\to 80}+\beta^{80}\cdot f^{40\to 80}+\gamma^{80}\cdot f^{20\to 80}$$

where *ASFF*-80 is the input sent to the detection head after the neck architecture; $\alpha^{80}$, $\beta^{80}$, and $\gamma^{80}$ are the feature matrices of the three source feature maps, respectively, which are the weights of each feature map. Those three feature weights are learnable parameters, and they represent the importance of each set of feature maps. The values of three feature matrices at a certain coordinate point satisfy α+β+γ=1. Moreover, three weights have been normalized and satisfy α,β,γ∈[0,1]. $f^{x\to 80}$ denotes the feature maps from the size of *x* to the size of 80. 

### 3.2. Attention Mechanism

The attention mechanism helps the network determine and detect the location and character of important features in the images; then, it assists in suppressing irrelevant or unimportant areas. This mechanism can help models in resisting interferences and improving the detection accuracies of the detectors. Since the introduction of the attention mechanism, it has made significant advancements in the field of computer vision. The attention mechanism has evolved from the early channel’s attention to the hybrid attention mechanism. Considering the fact that the contributions of different channels to tasks vary, the attention of the channel domain is to assign different weights to different channels and focus on those channels that are more critical to identify the specific objects. Spatial attention is about discovering the areas of interest in a task, which in most cases only occupy a small portion of the images. Then, it calculates attention weights through pixel similarities in context features. The hybrid domain attention uses both channel attention and spatial attention. Attention modules are the plug and play functional modules, commonly including CBAM attention [28], SimAM attention [29], and CoordAtt [30]. These three types of attention can be inserted into the network separately and compared. In our improved YOLOv8 network, the attention mechanism is placed at the end of the backbone structure.

#### 3.2.1. CBAM 

The CBAM (convolutional block attention module) is an influential representative of hybrid attention mechanisms. The CBAM has achieved excellent generalization enhancements in many past tasks. The CBAM is a lightweight attention module, which means it does not increase the parameters and computational power too much. This module consists of two independent sub-modules, named CAM (channel attention module) and SAM (spatial attention module), which are connected in series. The process can be expressed through the following equations:

(3) $$F_{C}=M_{C}\;\left(F\right)\;⊗\;F$$

(4) $$M_{C}=\sigma (MLP(Avg\left(F\right)+Max(F)))$$

(5) $$F_{S}=M_{S}\;\left(F_{C}\right)\;⊗\;F_{C}$$

(6) $$M_{S}=\sigma (f^{7\times 7}([Avg\left(F_{C}\right)\;;\;Max\left(F_{C}\right)]))$$

where F is the feature map input of the attention module; $F_{C},F_{S}$ operators are the output feature maps of the CAM module and the SAM module, respectively; $M_{C}$, $M_{S}$ are the convolutional computations in the channel attention module and the spatial attention module, respectively; *Avg* and *Max* are the average pooling and the max pooling operation, respectively; $f^{7\times 7}$ denotes a convolution with a kernel of 7 × 7; *σ* is the sigmoid function to normalize the attention weights; *MLP* is a multi-layer perceptron. Two pooled feature maps are concatenated together in Equation (6). The growth in the floating-point operations caused by adopting this attention module is very small.

#### 3.2.2. SimAM Attention

**Figure 4:**
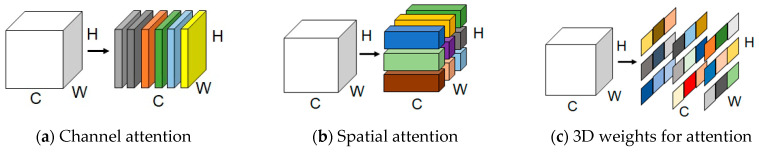
Comparisons of different dimensions’ weights.

SimAM attention is a representative of parameter-free hybrid domain attention mechanisms, which do not introduce extra parameters. It is designed based on neuroscience theories. Most hybrid attention mechanism operations are inherited into each individual block, which can only operate on features in one dimension of a channel or space. This results in differential treatments of the different channel contributions in channel attention but equal treatment of position information. Then, the characteristic of spatial attention is exactly the opposite. The absence of adaptabilities in cross-spatial and -channel variations was a drawback of past hybrid domain attention mechanisms. The SimAM module refines the 3D (three-dimensional) weights for points in each channel and each spatial local feature map region. The differences between setting the 3D weight approach by this mechanism and using the weight-generation method of existing hybrid domain attention mechanisms is shown in Figure 4.

The structure of existing basic attention modules often requires a series of complex operations, such as different kinds of pooling, convolutions, and fully connected layers. This module, proposed based on the consummate neural scientific theory, overcomes the above problem well. In neuroscience, informative neurons generally exhibit different firing patterns from adjacent neurons. Moreover, the activating neurons commonly suppress their adjacent neurons, leading to a phenomenon known as spatial suppression. Then, the neurons with spatial suppression effects have higher importance. Calculating the linear separability between a target neuron and other neurons is the simplest way to trace these neurons. Based on the above theory, an energy function for each neuron is proposed. The energy equation is expressed as:

(7) $$e_{t}\left(w_{t},b_{t},x_{i},y\right)={\left(y_{t}-\hat{t}\right)}^{2}+\frac{1}{M-1}\sum_{i=1}^{M-1}{\left(y_{o}-\hat{x_{i}}\right)}^{2}$$

where *t* is the target neuron; $x_{i}$ represents the other neurons in the same single channel; *M* is the quantity value of the channel; $w_{t}$ represents the weight; $b_{t}$ is the bias of the transform. See the following mathematical relations: $\hat{t}=w_{t}t+b_{t}$, $\hat{x_{i}}=w_{t}x_{i}+b_{t}$. From Equation (7), it can be seen that, when $y_{t}=\hat{t}$, $y_{o}=\hat{x_{i}}$, the energy function obtains the minimum value. Therefore, the process of solving the minimum of the equation is the key to realizing linear separability.

The lower value of the energy function means there is a greater difference between a neuron and other neurons on the same channel. From the perspective of computer vision, this neuron is more important. Therefore, the reciprocal of the energy function value can represent the importance of the neuron. The reciprocal of the energy function value is directly proportional to the criticality of this neuron. Equipping SimAM attention helps the network to improve the detection performance without adding any parameters, which leads to the generation of a lightweight, improved model. Furthermore, the architecture of SimAM attention is clear; its enhancement effect on small networks is more significant.

#### 3.2.3. CoordAtt 

CoordAtt (coordinate attention) is an attention mechanism that breaks the independence of channel and spatial attention modules and establishes cross-domain connections. Coordinate attention captures cross-channel information and embeds location information into the channels. In the past, the attention mechanism used local convolutions to extract the weights of spatial attention features, which could only capture local information and could not obtain global perceptions. Coordinate attention decomposes the channel attention information into two one-dimensional feature codes and aggregates the features along the horizontal and vertical directions. The CoordAtt module can capture remote associated characteristics in the one spatial direction and save accurate location information in another spatial direction; this helps the model to locate and identify targets more accurately. This attention module only adds a small number of parameters and is superior to dense detection tasks.

(8) $$T_{c}^{h}(h)=\frac{1}{W}\sum_{0\leq i<W}S_{C}(h,i)$$

(9) $$T_{c}^{w}(w)=\frac{1}{H}\sum_{0\leq j<H}S_{C}(j,w)$$

(10) $$g=\delta (O(\left[T_{c}^{h},T_{c}^{w}\right]))$$

(11) $$a^{h}=sigmoid(O^{h}(g^{h}))$$

(12) $$a^{w}=sigmoid(O^{w}(g^{w}))$$

where $T_{c}$ is the outlet of channel *c*; *h* and *w* are the height and width, respectively; *O* represents a shared 1 × 1 convolutional transfer function; δ is the nonlinear activate function; *sigmoid* is the sigmoid function; *a* is the attention weight value.

Compared to CBAM and SimAM, that calculate the importance of spaces and channels, the CoordAtt, which encodes precise position information, can better help the model locate the position of objects. X-ray security images show objects concentrated in suitcases, backpacks, security baskets, and other areas with a large amount of blank space; for these, the object position information in CoordAtt may have a greater impact on the sensitivity of detection.

### 3.3. Weighted Concatenate

Similar to the adaptive spatial feature fusion and attention mechanisms, the weighted concatenate connection is also a measure of endowing different weights to inputs from different contribution sources. In previous studies on network feature concatenation, researchers have not considered the importance of different approach features for the network but have instead merely integrated them equally together. The effective strategy is to give weights to these features according to their contributions of feature maps during feature fusion. When applied in the YOLOv8 network, this strategy is applied to feature concatenate connections in the pyramid. The feature concatenation pattern in the improved network uses the weighted option. This instrumental algorithm can be represented as:

(13) $$f^{out}=Concat(\frac{w_{1}\cdot f_{1}^{in}+w_{2}\;\cdot \;f_{2}^{in}}{w_{1}+w_{2}+e})$$

where $f_{1}^{in}$ and $f_{2}^{in}$ are the inputs of the feature concatenation; $f^{out}$ is the output; $w_{1}$ and $w_{2}$ are the learnable weights; e is a parameter to prevent instability, which is close to 0. The weighted concatenate method enables the network to have a more comprehensive understanding of feature maps. This weighted algorithm is a lightweight upgrade that promotes accuracy without burdening the network. In contrast to BiFPN [31], we have not changed the structure of the feature pyramid; this allows us to avoid introducing too many features to the feature fusion that have not yet been processed by the attention module.

### 3.4. Slide Loss Function

**Figure 5:**
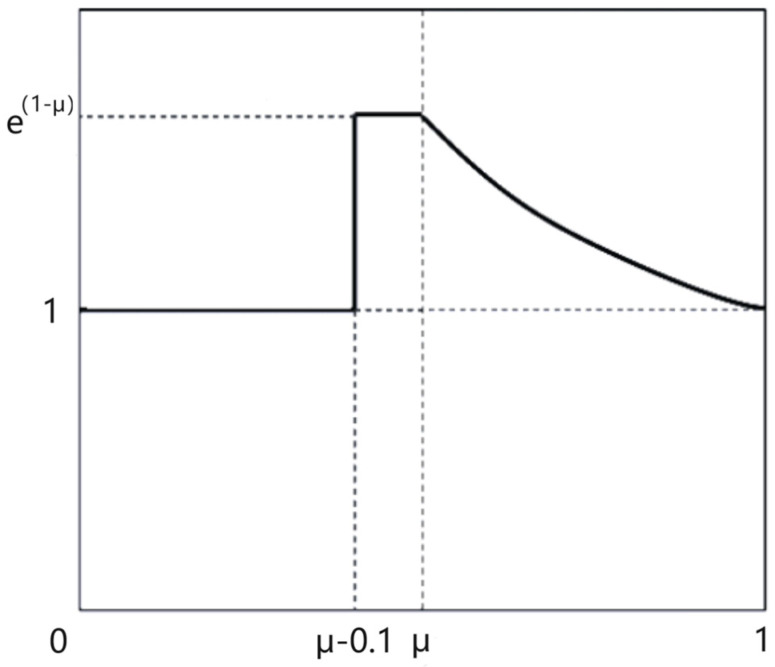
Graph of the slide loss function.

While the datasets in this paper are already very extensive and plentiful, there remain insufficiencies in using them to train a sophisticated model, which is due to the relatively small number of difficult samples. The backgrounds of the test sets comprise complicated environments, so there is a lack of robustness in these circumstances for the training features. The number of hard samples is lower than the requirement for training mature detection models. To address this sample imbalance, a weighted function was proposed [32]. The IoU value between the prediction box and the ground truth is the metric that should be used to distinguish between the simple samples and the hard samples. The threshold *µ* for discriminating samples is the average of the IoU values of all the bounding boxes. During the training and network learning, samples with an IoU value higher than the *µ* are simple samples; meanwhile, samples with predicted IoU values that are lower than the threshold are hard samples. Due to the poor ability to discern hard samples, there is a failure to effectively utilize instances during training. The role of the slide weight loss function is to assign a high weight value to hard samples and a low weight value to simple samples, so as to ensure that the loss function pays more attention to difficult samples. The assignment rule function is as follows:

(14) $$slide\left(i\right)=\left\{\begin{array}{l} \;1,\;i<\mu -0.1\\ e^{1-\mu },\;\mu -0.1<i<\mu \\ e^{1-i},\;i>\mu \end{array}\right.$$

where *slide*(*i*) is the slide function operation; *i* is the IoU between the prediction box and the truth in training; μ is the weight threshold. The function curve of the slide loss is shown in Figure 5. The solid line in the graph is the slide function curve, and the dotted lines mark the coordinate values. 

The slide loss function is introduced into the calculation of loss function, and the value of the BCE loss function for objection classification is weighted. The new total loss function is defined as:

(15) $$L_{total}^{*}=h_{BCE}*s{lide(l}_{BCE})+h_{CIoU}*l_{CIoU}+h_{DFL}*l_{DFL}$$

where $L_{total}^{*}$ is the total loss function that uses the slide loss; $h_{BCE}$ is the hyper-parameter for BCE loss; $l_{BCE}$ is the value of BCE loss; $l_{CIoU}$ and $l_{DFL}$ are the bounding boxes for regression loss; $h_{BCE}$,$\;h_{CIoU}$, and $h_{DFL}$ are set to 7.5, 0.5, and 1.5, respectively. Setting high weight coefficients near the threshold will increase the loss value of the instances that are difficult to classify, which allows the network to pay more attention to those instances. Since the weighted values in the formula are all greater than or equal to 1, the classification loss becomes larger; this means that the role of classification in the overall network is larger. Moreover, the gradient decline of loss function becomes slower, so it may require that the number of training epochs is higher.

## 4. Experiment and Analysis

**Figure 6:**
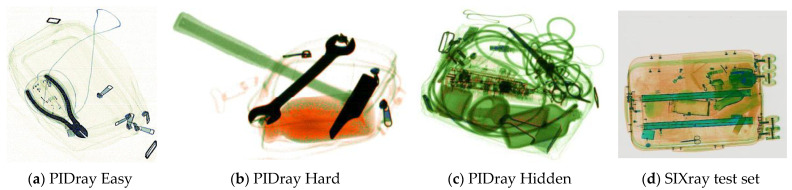
Examples of four test sets: (**a**) image from PIDray Easy test set; (**b**) image from PIDray Hard test set; (**c**) image from PIDray Hidden test set; (**d**) image from SIXray test set.

In this section, some experiments are conducted on PIDray and SIXray test sets to evaluate the effectiveness of the modified models. In Section 2.1, the particulars of the three test sets in PIDray dataset were explained. Examples of X-ray contraband images, from the PIDray Easy, PIDray Hard, PIDray Hidden, and SIXray test sets, are shown in Figure 6.

### 4.1. Experimental Configuration 

All experiments are conducted in the same environment with Ubuntu system, CUDA v10.2, three GeForce RTX 2080Ti GPUs, 36GB memory, and Python v3.8. The image height and width are set to 640 × 640, respectively. The train epoch of YOLOv8 model is set to 300. Due to the slow convergence, owing to the loss value increase caused by slide function, the epoch is set to 450 when the slide function is used. The probability hyperparameter of Mosaic data augmentation is set to 1.0, and the probability coefficient of mix-up data augmentation is set to 0.5. From the SIXray dataset, there are 7496 images in the training set and 1433 images in the test set, without the many background images.

### 4.2. Evaluation Metrics

The mAP (mean average precision) is commonly used as the standard measure of detector performance in detection tasks. The mAP is selected as the metric of these modified experiments of the X-ray security inspection algorithm. The mAP is the mean value of the AP (average precision), and the AP of a model consists of two aspects: precision and recall in detecting. There are four symbols to define them: TP means positive samples predicted as positive; TN means true negative samples, which are negative predicted as negative; FP means false positive samples, which are negative predicted as positive; FN means false negative samples, which are positive predicted as negative.

(16) $$Precision=\frac{TP}{TP+FP}$$

(17) $$Recall=\frac{TP}{TP+FN}$$

In Equation (16), the mathematical expression TP+FP denotes all positive samples detected; Precision means the proportion of true samples among all positive samples detected; Recall denotes the probability of searching for full positive samples.

(18) $$mAP=\frac{1}{M}\sum_{n=1}^{M}\int_{0}^{1}P_{i}(R_{i})dR_{i}$$

The setting of different thresholds results in different precision values and recall rates. The curve that uses precision and recall as the vertical and horizontal coordinates is called the PR curve. The integral of the PR curve is the AP value, and the mean value of all categories of AP values is mAP. The mAP is calculated as Equation (18). The character *M* denotes the number of categories. The rear integral in Equation (18) is the AP value of one category. The mAP_50_ is the average precision when the IoU threshold is set to 0.5, while mAP_50:95_ refers to the mean value of ten accuracies when the IoU threshold is set from 0.5 to 0.95 with a step size of 0.05.

### 4.3. Soft-NMS 

NMS (non-maximum suppression) is a major post-processing section in target detection. But NMS has a flaw: for candidate boxes with overlaps, if the IoU values of the box with the highest confidence score and others that intersect with it are more than the threshold, they will be deleted. Meanwhile, the boxes below the threshold are retained. When the model detects the serried and occluded scenes, this drawback will be magnified. The detection of objects that are obstructed by other objects or backgrounds will be suppressed under the previous non-maximum suppression regulation. 

Bodla et al. [33] proposed a Soft-NMS algorithm to avoid the low recall caused by certain image environments. The soft non-maximum suppression does not involve the rough deletion of detection boxes whose IoU are too large; instead, it involves assigning weighted values to all boxes except for the ones with the best scores. Those weights will reduce the confidence scores of the boxes. The weights will attenuate the scores of adjacent detection boxes that coincide with the one with the highest score. The more boxes coincide with the detection box, the more severe the score attenuation. After that, the predictor uses the confidence threshold to filter the boxes. The attenuation function is a gaussian function:

(19) $$Soft\left(i\right)=Confidence(i)e^{-\frac{{IoU(H,b_{i})}^{2}}{\sigma }}$$

where *Confidence*(*i*) is previous confidence score value of the box; *H* is the prediction box with the highest score; $b_{i}$ is another box that overlaps with *H*; σ is the attenuation coefficient. The computational complexity of Soft-NMS is the same as the traditional methods, and its computational cost is petty. Soft-NMS can significantly improve the detection performance of dense and occluded targets.

### 4.4. Data Augmentation

Data augmentation is a commonly used technique in computer vision tasks. Training set scenes generally do not fully include the scenes for testing. Although the datasets in this paper already have large content, more dataset instances will benefit from training the model while ensuring good sample quality. When data are insufficient, data augmentation fulfills the need to generate more training data from existing training sample data. The objects in the new images, transformed by data augmentation, may be placed in any position or direction in the image. The objects in the X-ray security datasets are also arranged in a disorderly manner, and these X-ray images with the features are visually similar to those data-enhanced images. Furthermore, data augmentation also constructs occlusion shapes between objects, just like in X-ray security inspection data images. In the YOLO series of detectors, there are several main data augmentation methods: mosaic [34] data augmentation, mix-up [35] data augmentation, and copy–paste [36] data augmentation. Cutmix [37] data augmentation is another available option for image data enhancement in object detection. The copy–paste method is used for instance segmentation.

The specific implementation of mosaic data enhancement is to splice four training set images into one image; this forms a new training sample. These four images are combined after random scaling, cropping, and layout operations. The proportion of the four images to the total image is also random in each mosaic enhancement operation. After the mosaic operation, the true value box of the positive sample will also change accordingly. The four images constitute a batch. During the batch normalization process, the data from four images are calculated simultaneously. Therefore, the batch size does not need to be set too large. The mosaic algorithm enhances the generalization ability of the models and sharply improves the detection ability for occluded and concealed objects.

Mix-up data augmentation is applied by being added to the mosaic algorithm. The core principle of mix-up augmentation is to linearly stack two different images in a certain proportion to create a new sample, and the labels of the two images are also linearly composed. Mix-up makes further efforts to expand the diversity of datasets and, to some extent, prevents overfitting of the training set; this is similar to other data augmentation approaches. The visual effect of mix-up data augmentation is to map two images in different shades onto one image, which includes instances of two images. This overlapped appearance through mix-up mapping looks like the occlusion that occurs in X-ray images; this is not a case of one object blocking a part of another object. The occlusion in X-ray datasets generally performs as a form of deep shade, with overlapping occlusions appearing darker and vaguer than their non-occluded counterparts. The images expanded by the mix-up method are similar to those used in occlusion detection, and the mix-up method manifestly improves the de-occluded learning ability of network.

**Figure 7:**
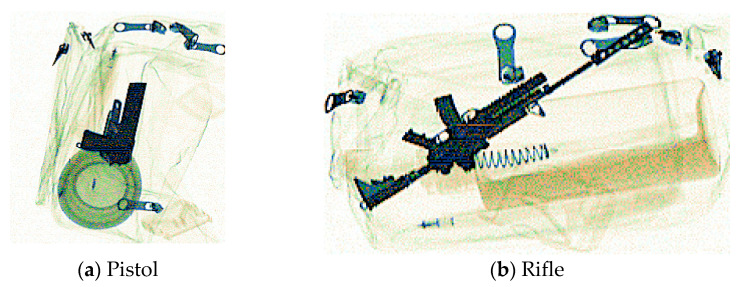
Different types of guns in the gun category on PIDray dataset: (**a**) an example of a pistol; (**b**) an example of a rifle.

As shown in Figure 7, using the PIDray dataset, the samples in the gun category (including rifles, pistols, and revolvers) have apparent distinction in shape and size. Therefore, it is difficult for the network to learn the gun category comprehensively; this gives rise to a low recall rate for the gun category in the original models. To boost the check performance of guns with poor recall rate, the following data modifications have been made. There is an adjustment to the ratio of the training images containing guns which expands it by four times, making it more probable to be sent into network learning. On the SIXray dataset, the recall value of the gun categories exceeds the overall recall rate, so there is no adjustment for the ratio of gun instances.

### 4.5. Results and Evaluation

In this section, the modified methods are used in experimentation using the YOLOv8 model. The detection models are used for the detection of prohibited items using X-ray to verify the effectiveness of the improved methods on PIDray dataset and SIXray dataset. After utilizing the YOLOv8n model with all the modified algorithms, the verification results of the three attention modules mentioned in Section 3.2. are compared to identify the optimal balanced model for use with PIDray test sets. An evaluation and analysis of the model improvement performance is laid out in the following, utilizing the three types of attention module for contraband detection using the PIDray dataset. The mAP numerical comparison is displayed in Table 1.

**Figure 8:**
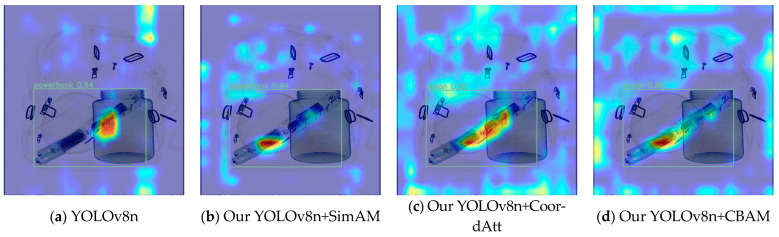
Comparisons of baton instance feature heat maps between original YOLOv8n and improved YOLOv8n with different attention modules.

**Figure 9:**
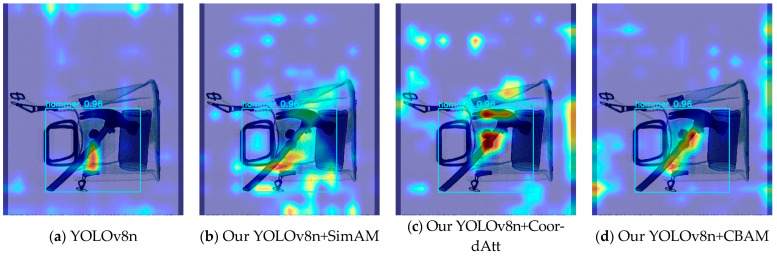
Comparisons of hammer instance feature heat maps between original YOLOv8n and improved YOLOv8n with different attention modules.

The three improved YOLOv8n models mentioned above are based on the employment of all the improved algorithms; note, however, that the embedded attention modules are different. The three improved models all have superior mAP values compared to the original YOLOv8n model on the three test sets. Among them, using the Easy test set, the most effective progress was made by the improved YOLOv8n model that incorporated CoordAtt; this achieved a 2.7% increase in mAP. The model-embedded CoordAtt also achieved the best detection performance outcome on the Hard test set, achieving an increase of 3.1%. On the Hidden test set, with heavily obstructed and hidden prohibited objects, the improved YOLOv8n model using SimAM achieved higher indicators in detection performance than the other two models, improving YOLOv8n’s benchmark accuracy by 9.8% for occluded and hidden objects. Partly due to its low benchmark value on the Hidden set, the extent of increase is sharper. Compared to the above models, the accuracy score achieved by the model with CBAM is inferior to that with the CoordAtt module in all aspects. But on the Easy test set, it achieved a score which was 0.2% higher than the model using SimAM. Comparing the results of the SimAM and CoordAtt modules in the model, the use of CoordAtt achieved mAP values of 90.2% and 90.5% on the Easy and Hard datasets, respectively; these results are 0.6% and 0.5% higher than those achieved by the model using SimAM. This analysis indicates that, on the basis of the YOLOv8n model, CoordAtt is more successful in simple X-ray contraband-detection scenarios. However, on the Hidden test set, the mean accuracy of the instance utilizing CoordAtt was 0.5% lower than that achieved by the use of SimAM. Due to the relatively small difference in the enhancement of attention between the SimAM and CoordAtt models on the Hidden dataset, and the fact that the overall testing behavior of one model on the three datasets will be more important, CoordAtt was ultimately selected as the optimal choice. The visual comparisons of the activation features of the three types of attention are shown in Figure 8 and Figure 9.

In Figure 8 and Figure 9, the brighter and redder area indicates that the feature information of its heat area is more significant for prediction. The attention module is applied to the deep convolutional features after SPPF in the network, so the feature heatmap is also applied to the deep convolutional feature layers that are the ones that were input into the detection head. The localization information of deep activation features is often concentrated at the center of the box that plays a decision-making role in model prediction. From the above figures, it can be seen that the models using CoordAtt, encoded with accurate positional information, achieved a redder center in the heat map. In Figure 8, the YOLOv8n model and the YOLOv8n model embedded with SimAM mistakenly predict a baton to be a powerbank. From the comparisons, it can be seen that the hot region of the heat map of the models embedded with the attention options is closer to the intact contour of the target. In addition, the model that applies CoordAtt has a larger heat region and brighter color. These examples, to some extent, provide evidence that the model embedded with CoordAtt have superior accuracy in comparison with the other options.

Table 2 shows the ablation experiment results of all the algorithms on the YOLOv8n model. Compared to the raw model, all improved algorithms have almost no increase or decrease in the number of model parameters, so the numbers of parameters are not listed. It can be seen that applying all modified algorithms together is effective. Firstly, when only data augmentation was used, the model achieved mAP_50_ values of 88.3%, 88.9%, and 74.1% on the Easy, Hard, and Hidden test sets, respectively. This model has dramatically improved its accuracy compared to the original YOLOv8n model, especially with a 4.3% improvement in application to the Hidden test set. From this, it can be seen that data augmentation plainly improves the generalized detection ability of the network. Data augmentation has created many scenes with obstructed and hidden prohibited objects for training, which has greatly improved the model’s ability to detect obstructed and hidden objects. Therefore, the subsequent improvements are all based on the use of data augmentation. Secondly, the further addition of the Soft-NMS algorithm also increased detection performance on three test sets, leading to a 4.2% improvement on the Hidden dataset to achieve an mAP_50_ of 78.3%. For the convenience of conducting ablation experiments, ASFF was combined with weighted concatenate to verify. It can be seen that, after adding ASFF + weighted concatenate, the mAP_50_ values of the model reached 88.6%, 90.3%, and 78.3%, which decreased on the Easy dataset, increased by 0.2% on the Hard dataset, and remained unchanged on the Hidden dataset. Next, the ablation study concerns the approach involving ASFF + weighted concatenate, the CoordAtt module, and the slide loss function; this only uses two to test the ablation model that lacks one algorithm. The models obtained in the absence of one of the above algorithms were not found to be superior to the final model. Only the combination algorithm without CoordAtt achieved an mAP_50_ of 79.1% on the Hidden dataset; this is the same as the model’s precision when applying all algorithms, but it performs poorly on the Easy and Hard datasets. Moreover, there is no overall decrease in mean accuracy among the three datasets after using a new algorithm compared to not using it. The results show that the improvement achieved when using all algorithms is efficacious, with the best mAP_50_ and mAP_50:95_ values reaching 90.2%, 90.5%, 79.1%, 81.8%, 80.5%, and 67.9%, respectively. Considering the impact of increasing the number of training epochs using the slide loss function, an experiment that only increases the number of training epochs without using the slide loss function was conducted. The mAP_50_ values reached 89.3%, 90.1%, and 78.6%, respectively. This indicates that the slide loss function is effective. On the Hard set of the PIDray dataset, data augmentation and Soft-NMS have a dominant outcome for model performance improvement.

**Figure 10:**
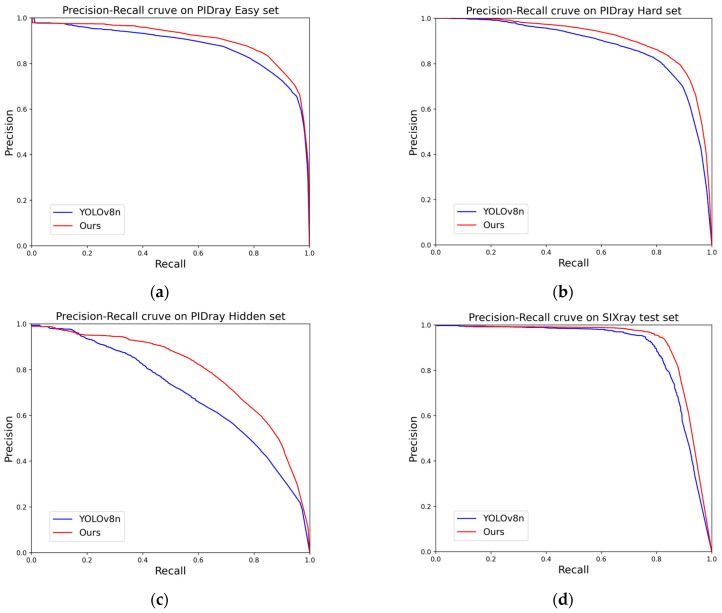
Comparisons of PR curve: (**a**) PR curve on PIDray Easy test set; (**b**) PR curve on PIDray Hard test set; (**c**) PR curve on PIDray Hidden test set; (**d**) PR curve on SIXray test set. (**a**) Comparison between YOLOv8n and YOLOv8n with our method on the Easy set of the PIDray dataset. (**b**) Comparison between YOLOv8n and YOLOv8n with our method on the Hard set of the PIDray dataset. (**c**) Comparison between YOLOv8n and YOLOv8n with our method on the Hidden set of the PIDray dataset. (**d**) Comparison between YOLOv8n and YOLOv8n with our method on the SIXray test set.

The checkmark in the tables represent that the model applied this algorithm. The precision values in Table 3 are calculated using mAP_50_. As can be seen from the Table 3, on the final model, the gun and plier categories did not achieve the highest precision. But the overall accuracy of the model using all algorithms was optimal, and a mAP of 91.4% was achieved on the SIXray test set. Compared to the original YOLOv8 model, the ultimate improved YOLOv8n model has a 2.4% increase in the mean accuracy of all types. Specifically, the mAP values of the five types were increased by 4.1%, 1.6%, 3.1%, 0.6%, and 2.9%, respectively. In the ablation experiments, the highest mAP value of the gun category was only 0.1% higher than that achieved by the model using all of the algorithms. In this case, the Soft-NMS algorithm instead suppressed the detection of the pliers, reducing the accuracy by 1.0%. On the SIXray datasets, the improvement effect of the slide loss function is relatively significant. All the models that have applied the slide loss function obtained accuracy values above 90.0 mAP, and the highest accuracy values of each category were obtained using the models that adopted this function. The increased extent on the SIXray dataset that was achieved through data augmentation and the Soft-NMS algorithm is weaker than that achieved through the PIDray dataset. The comparisons of the PR curves between the improved YOLOv8n model and the raw YOLOv8n model are shown in Figure 10.

The larger the integral of the PR curve, the higher the mAP value, indicating that the improved method has varying degrees of improvement effect on all four test sets. Observing the PR curve comparison graph, it can be concluded that the improved model’s PR curve is generally above that of the unimproved model. Although there is a little segment of low recall and high precision in the curve on the Easy and Hidden dataset graphs, the overall integration areas of the four pairs of the PR curves are larger. This means that the improvement effect is valid for the X-ray security inspection of prohibited items. The ability of the network to recognize occluded and hidden security prohibited items has been improved even more on the Hidden test images.

Table 4 shows the comparison results of different advanced detection methods using the PIDray dataset to verify the progressiveness and effectiveness of the here-proposed models. In testing results conducted by Zhu et al. [15], Wang et al. [16], Song et al. [21], and Cheng et al. [24], the accuracy of the performances of detectors such as FCOS, Faster R-CNN, Mask R-CNN, and SSD are not good. All of the existing algorithms mentioned show worse performances than the proposed models in the above papers. From Table 4, it can be seen that the proposed models based on the YOLOv8 models perform best using the PIDray dataset. 

On account of the different partitions of the training and testing sets, the comparability of results from different studies is not consistent. Only other advanced models with the same dataset division as this paper have been compared. From Table 5, Yolov7-tiny algorithm has low accuracy on both datasets. YOLOv5s has a 1% higher accuracy than the original YOLOv8n model on the SIXray dataset, while YOLOv8n has fewer parameters. Ma et al. [38] proposed the POD-Y model, which has a higher degree of accuracy than many existing models, but our methods achieve higher detection accuracies than POD-Y while sharply reducing the number of model parameters. Our YOLOv8n and YOLOv8m models achieved increasements of 2.4% and 1.8%, resulting in mAP values of 91.4% and 93.3%, respectively. The improved algorithms used in this paper hardly affect the number of model parameters, and even slightly decreased on the medium models. Among the algorithms in this article, the ones that will affect the model parameters are ASFF, weighted concatenate, and CoordAtt. Meanwhile, all three are lightweight improvements that retain the parameter count of the original model. Lightweight models do not occupy too much memory, do not add too much computational burden, and have a fast inference speed. This means that they provide practical convenience for real-world hardware devices, which have poor image processors and low memory capacity. They also offer the possibility of meeting real-time detection requirements using low-cost devices. The improved lightweight YOLOv8n model has the smallest number of parameters, but its accuracy has exceeded all the other algorithms using the PIDray and SIXray datasets. The improved YOLOv8m models with larger numbers of parameters have higher accuracy performances. The models proposed in this paper achieved a state-of-the-art accuracy using the PIDray and SIXray datasets.

## 5. Discussion

Enhancement of receptive field and enhancement of feature extraction are the major tacks in the modification of existing detectors. ASFF structure and weighted concatenate connection are used to refine and efficiently utilize features from existing data. Attention helps the model identify the key points of the images in channel and spatial domains. A comparative study was conducted on SimAM, CoordAtt, and CBAM to select the attention that is suitable for the network. There are always some instances in the datasets that are easy to learn and predict, while others are the opposite. Slide loss function is a simple method to help a network to strengthen the learning of difficult samples. The above lightweight improvements help the network learn features more finely during training and have resulted in high-precision and lightweight detectors.

Data augmentation has been used to produce some images that were not originally present in the dataset. These images have similar characteristics to those in the original dataset. However, they additionally have many occlusions and overlapping placements added, which helps the model improve in learning to identify occluded prohibited objects. Soft-NMS provides an improvement on the non-maximum suppression operation. It adds a mathematical operation with a small amount of computation, making it easier for the network to identify occlusion and hide contraband. These two algorithms have profoundly raised our accuracy in occluded scenes.

In addition, our method has an almost negligible impact on the number of model parameters. But there are still some limitations to this study. The detection accuracies of the three categories of lighter, knife, and gun in the PIDray dataset are poor, which brings down the overall detection accuracy. Meanwhile, the detection performance of the Hidden test set for severely occluded scenes has not yet reached the same level as that of unobstructed detection. Furthermore, this paper does not cover the false alarms which occur in real-world systems. During the experiment, it was found that the NWD (normalized Wasserstein distance) [39] algorithm had no positive effect on this model. The NWD algorithm is a measure that is only effective for detecting small targets, and many experiments have shown that its effects on large and medium targets are not as good as those achieved using the IoU. The improvement in the accuracy of NWD for small targets is less than its reduction for other scales, resulting in an overall performance deviation of NWD on the model. Thus, the final network algorithm did not adopt it.

## 6. Conclusions and Future Work

This paper mainly proposed a lightweight and high-precision detection method for X-ray security inspection, which also achieved successful performance in the detection of obscure and hidden prohibited objects. 

To demonstrate the effectiveness of those algorithms, many studies were conducted. On PIDray and SIXray test datasets, our proposed YOLOv8n model achieved mAP_50_ values of 90.2%, 90.5%, 79.1%, and 91.4%, respectively; these are 2.7%, 3.1%, 9.3%, and 2.4% higher than those achieved using the original model. The model has a small number of parameters and low inference time; these features are advantageous for hardware devices. Specifically, the proposed YOLOv8m model achieved accuracies of 92.9%, 92.7%, 82.9%, and 93.3%, respectively; these are 2.6%, 1.9%, 9.4%, and 1.8% higher than those achieved by the original model, respectively. The proposed models have high accuracies for X-ray security detection and good recognition ability for severely occluded objects. Moreover, the numbers of proposed model parameters are only about 3 million and 25.8 million, respectively. The proposed method is a practical way to handle contraband detection in complex field scenarios.

In future work, the model will be optimized to improve the performances of specific classes with poor accuracy. The train set and test set will be further expanded to validate the model’s generalization ability and robustness. Moreover, in reality, false alarms in detection devices are inevitable and a low false alarm rate is required. Therefore, the next step can be to conduct further research on these issues.

## Figures and Tables

**Table 1:** Comparison of performance between improved models of different attention modules.

Model/TestSet	$\mathrm{Easy}/{mAP}_{50}$	$\mathrm{Hard}/{mAP}_{50}$	$\mathrm{Hidden}/{mAP}_{50}$
YOLOv8n	87.5	87.4	69.8
our YOLOv8n+SimAM	89.6	90.0	**79.6**
our YOLOv8n+CoordAtt	**90.2**	**90.5**	79.1
our YOLOv8n+CBAM	89.8	89.9	78.8

**Table 2:** Ablation experiments for all algorithms on PIDray dataset based on YOLOv8n model.

Method					$\mathrm{Test}\;\mathrm{Set}/{mAP}_{50}$			$\mathrm{Test}\;\mathrm{Set}/{mAP}_{50:95}$		
Data Augmentation	Soft-NMS	ASFF + Weighted Concatenate	CoordAtt Attention	Slide Function	Easy	Hard	Hidden	Easy	Hard	Hidden
					87.5	87.4	69.8	77.8	73.2	52.1
√					88.3	89.9	74.1	79.0	77.0	61.4
√	√				89.0	90.1	78.3	80.4	78.9	66.2
√	√	√			88.6	90.3	78.3	80.6	79.2	66.3
√	√	√	√		88.2	90.1	78.9	80.0	79.5	67.1
√	√	√		√	88.7	89.9	**79.1**	80.4	79.5	67.6
√	√		√	√	88.3	90.2	78.0	80.1	79.7	66.6
√	√	√	√	√	**90.2**	**90.5**	**79.1**	**81.8**	**80.5**	**67.9**

**Table 3:** Ablation experiments for all algorithms on SIXray dataset based on YOLOv8n model.

Method					$\mathrm{Category}/{mAP}_{50}$					
Data Augmentation	Soft-NMS	ASFF + Weighted Concatenate	CoordAtt Attention	Slide Function	All	Knife	Gun	Wrench	Pliers	Scissors
					89.0	85.7	91.2	84.3	91.6	92.1
√					89.8	89.2	92.3	84.4	91.4	91.7
√	√				89.6	89.9	92.0	84.7	90.5	91.8
√	√	√			89.9	88.2	92.2	85.7	91.3	92.2
√	√	√	√		89.7	89.0	92.6	84.2	90.7	91.9
√	√	√		√	90.8	**89.8**	92.7	87.3	92.3	91.8
√	√		√	√	90.5	89.7	**92.9**	85.7	91.6	92.5
√		√	√	√	91.1	88.8	92.1	86.9	**93.2**	94.3
√	√	√	√	√	**91.4**	**89.8**	92.8	**87.4**	92.2	**95.0**

**Table 4:** Comparison of different detection algorithms on PIDray dataset.

Model	Year	Easy	Hard	Hidden	Params/M
AMOD-Net [15]	2021	86.7	88.3	66.9	-
SDANet [16]	2021	71.2	64.2	49.5	-
YOLOv5s	2020	85.2	86.9	61.5	7.04
Song et al. [21]	2022	88.2	89.2	70.5	5.67
YOLOv7-tiny	2022	63.4	57.3	53.2	6.04
Cheng et al. [24]	2023	84.5	65.4	49.2	112.6
YOLOv8n	2023	87.5	87.4	69.8	3.008
YOLOV8n+ours	2023	90.2	90.5	79.1	3.014
YOLOv8m	2023	90.3	90.8	73.5	25.857
YOLOv8m+ours	2023	92.9	92.7	82.9	25.824

**Table 5:** Comparison of different detection algorithms on SIXray dataset.

Model	Year	All	Knife	Gun	Wrench	Pliers	Scissors	Params/M
YOLOv5s	2020	90.0	85.2	92.0	87.5	91.8	93.4	7.024
YOLOv7-tiny	2022	82.6	70.2	89.6	78.9	87.3	87.1	6.018
POD-F-R [38]	2022	86.1	92.5	88.9	83.8	87.6	87.7	118.32
POD-F-X [38]	2022	86.9	82.6	90.0	84.1	88.2	89.6	119.67
POD-Y [38]	2022	90.4	87.9	92.6	87.6	92.1	91.8	47.19
YOLOv8n	2023	89.0	85.7	91.2	84.3	91.6	92.1	3.007
YOLOV8n+ours	2023	91.4	89.8	92.8	87.4	92.2	95.0	3.013
YOLOv8m	2023	91.5	88.8	92.5	88.2	94.6	93.5	25.843
YOLOv8m+ours	2023	93.3	90.8	94.3	91.6	94.2	95.4	25.821

## Data Availability

The dataset is available at https://github.com/bywang2018/security-dataset, 2021.

## Abbreviations

YOLO	you only look once
ASFF	adaptive spatial feature fusion
CoordAtt	coordinate attention module
NMS	non-maximum suppression
mAP	mean average precision
EDXRD	energy dispersive X-ray diffraction
SVM	support vector machine
bAP	base average precision
R-CNN	region-convolutional neural network
SSD	single shot multibox detector
MSA_1_	multi-scale attention architecture
MSE	multi-scale feature extraction
PMMW	passive millimeter wave
PANet	path aggregation network
MSA_2_	multi-scale smoothed atrous
MCA	material-aware coordinate attention
IoU	intersection over union
SD-SIoU	shape decoupled SIoU
EFE	edge feature extractor
MFE	material feature extractor
FPN	feature pyramid network
DOAM	de-occlusion attention module
ELAN	efficient layer aggregation network
SPPF	spatial pyramid pooling -fast
PAN-FPN	path aggregation network FPN
BCE	binary cross entropy
DFL	distribution focal loss
CIoU	complete-IoU
CBAM	convolutional block attention module
CAM	channel attention module
SAM	spatial attention module
3D	three-dimensional
NWD	normalized Wasserstein distance
